# Evidence for two types of nrDNA existing in Chinese medicinal fungus *Ophiocordyceps sinensis*

**DOI:** 10.3934/genet.2017.3.192

**Published:** 2017-08-25

**Authors:** Chih-Sheng Chen, Ching-Tsan Huang, Ruey-Shyang Hseu

**Affiliations:** Institute of Microbiology and Biochemistry, College of Life Science, National Taiwan University, Taipei, Taiwan (R.O.C)

**Keywords:** *Ophiocordyceps sinensis*, nrDNA, species complex, cryptic species

## Abstract

Nuclear ribosomal DNA (nrDNA) sequences are widely used in the molecular classification of fungi. Previous phylogenetic studies of highly-valued traditional Chinese medicinal fungus *Ophiocordyceps sinensis* were mostly based on 18S and internal transcribed spacer (ITS) regions (ITS1, 5.8S and ITS2) of nrDNA. However, the disparity manifest in the low sequences identities between different *O. sinensis* isolates has led to argumentative hypotheses for this phenomenon, such as the “species complex” or “cryptic species” hypotheses. In the present study, four types of nrDNA (GC, AT-1, AT-2, and T) were identified using four primer pairs to amplify the nrDNA of six *O. sinensis* isolates. We demonstrate that each *O. sinensis* isolate contained two types of nrDNA, the omnipresent GC-type and a coexistent type alternating between the remaining three. This crucial discovery challenges the established notion of one type of nrDNA per species. We therefore propose that the composition of nrDNA types should be taken into consideration in studies of fungal genetics and classification.

## 1. Introduction

*Ophiocordyceps sinensis* (Berk.) Sacc. is a typical entomogenous fungus found in southwestern China. It grows parasitically on the larvae of Lepidoptera, particularly those belonging to the genus *Hepialus*. In Asia, *O. sinensis* is considered to be a valuable traditional fungus that has several medicinal effects [1].

Using phylogenetic trees based on the ITS region of nrDNA sequences (ITS1, 5.8S and ITS2 nrDNA sequences), Kinjo and Zang [2] suggested that the 17 collected *O. sinensis* isolates from 11 southwestern China localities could be divided into two subgroups. Stensrud et al. [3] analyzed ITS region nrDNA variation among 71 sequences of *O. sinensis* available in the EMBL/GenBank databases, and suggested that *O. sinensis* isolates could be divided into three subgroups and that “cryptic species” existed within the *O. sinensis* grouping. Whether it is the “subgroup” concept or “cryptic species” concept, low sequences identities are not normally observed in the *Cordyceps/Ophiocordyceps* genus.

In the present study, we designed four primer pairs to amplify the partial 18S and ITS region nrDNA sequences from six *O. sinensis* isolates. We have demonstrated that a single *O.* *sinensis* isolate could simultaneously have two types of nrDNA sequences.

## 2. Materials and Methods

### 2.1. Fungal Specimens and Strains

The specimens and strains used in this study are listed in Table 1. Strains were cultured in 250 mL potato dextrose broth (PDB; DIFCO, Detroit, MI, USA) in 500 mL flasks agitated at 100 rpm at 10 °C. The mycelia were harvested after 8 weeks and washed with sterile water. All specimens and mycelia were then lyophilized and stored at −20 °C prior to further analysis.

**Table 1.** *Ophiocordyceps sinensis* isolates examined in this study.

Isolate No.	Tissue	Locality	Primer pair	Product size (bp)	Accession No.	nrDNA Type
Cs7528A-0901	Stroma	Tibet, China	NS5/GCITS4	1219	AM931048	GC
			NS5/AT12ITS4	1223	AM931055	AT-2
Korea-0901	Stroma	Qinghai, China	NS5/GCITS4	1219	AM931050	GC
			TITS5b/TITS4	477	AM931059	T
W1023-0901	Stroma	Sichuan, China	NS5/GCITS4	1219	AM931049	GC
			TITS5b/TITS4	477	AM931060	T
RS2-0901	Mycelia	Tibet, China	NS5/GCITS4	1219	AM931051	GC
			NS5/AT12ITS4	1219	AM931057	AT-1
RS3-0901	Mycelia	Tibet, China	NS5/GCITS4	1219	AM931052	GC
			NS5/AT12ITS4	1223	AM931056	AT-2
RS4-2-0901	Mycelia	Tibet, China	NS5/GCITS4	1219	AM931053	GC
			TITS5b/TITS4	477	AM931058	T

### 2.2. DNA Preparation

DNA was isolated as described by Moncalvo et al. [4]. Briefly, the ground sample (60 mg) was transferred to a 1.5 mL microcentrifuge tube containing 600 µL of lysis buffer (50 mM Tris-HCl, 50 mM EDTA, 3% SDS, and 1% 2-mercaptoethanol at pH 7.2). The tube was incubated in a water bath at 65 °C for 1 h, and the aqueous phase was then extracted twice using 600 µL of PCI (phenol/chloroform/isoamyl alcohol = 25:24:1; Sigma Co., Saint Louis, MO, USA). After extraction, the aqueous phase was transferred to a new tube, and the precipitated DNA was mixed with 0.1 volumes 3 M sodium acetate to 0.6 volumes isopropanol. The DNA was centrifuged at 15 000× *g* for 5 min, washed twice with cold 70% ethanol and dried for 30 min in a vacuum oven at 37 °C. The remaining DNA pellet was then resuspended in 100 µL of TE buffer (10 mM Tris-HCl and 1 mM EDTA; pH 8.0) containing 2 µL of RNase (500 µg/mL; Roche Applied Science Co., Mannheim, Germany) and incubated in a water bath at 37 °C for 1 h. After the addition of 100 µL of chloroform, the aqueous phase was transferred directly into a new tube. DNA was precipitated using 0.1 volumes 3 M sodium acetate to 0.6 volumes isopropanol and then again centrifuged at 15 000× *g* for 5 min. The DNA was resuspended in 100 µL sterile water and stored at −20 °C.

### 2.3. PCR Amplification

**Figure 1.**
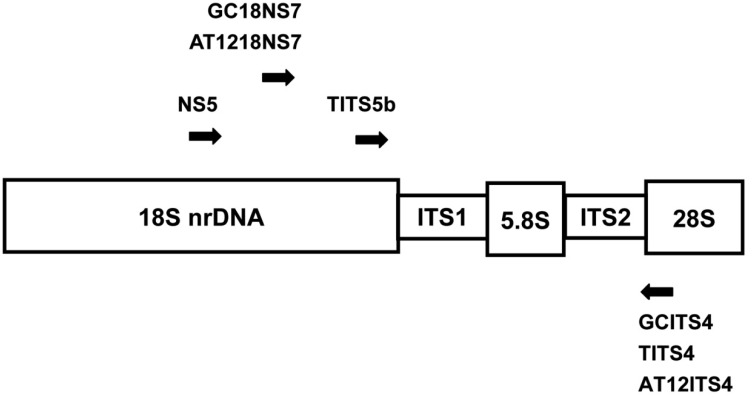
Locations on nrDNA of PCR primers designed in the present study. Arrowheads represent the 3′ end of each primer.

The primers were designed according to White et al. [5] and Kinjo and Zang [2]. The primers sequences and locations used in the present study are given in Table 2 and Figure 1. Amplifications were carried out using a Perkin-Elmer thermocycler 480 (Applied Biosystems, Foster City, CA, USA) in 25 µL reaction mixture containing 50 ng template DNA, 2.5 µL 10× PCR buffer (ProTech Professional Technical Services, Inc., Pittsburgh, PA, USA), 0.2 mM of each dNTP, 0.5 µM of each primer, and 0.625 U of super taq DNA polymerase (ProTech Professional Technical Services, Inc., Pittsburgh, PA, USA). Amplifications were performed using the following PCR program: initial denaturation at 96 °C for 2 min, followed by 35 cycles at 96 °C for 45 s, 54 °C for 45 s, 72 °C for 2 min, and a final extension step at 72 °C for 10 min. A negative control (dsH_2_O) was included.

Gel electrophoresis of PCR products was performed on a 1.2% agarose gel containing 2 mL ethidium bromide per 100 mL TAE buffer (0.02 M Tris base, 0.02 M acetate and 0.0005 M EDTA, AMRESCO Co., Solon, Ohio, USA). Gels were visualized using an UV-trans-illuminator.

**Table 2.** Primers used in this study [2],[5].

Primer	Primer sequence (5′→3′)
NS5	AACTAAAGGAATTGACGGAAG
GCITS4	ATCCGAGGTCAACTGGAGGGTGTG
AT12ITS4	GCTTCCGGTGCGAGGTTCTCGGTG
TITS4	AGTGCGAGGTTCTTAGTAAGCTATTGCG
TITS5b	TCGAGTTACCACTCCTAAACCCCCTGC
GC18NS7	TCCGGCAGTGCGCCGGCTTC
AT1218NS7	CGTACTACTCTAGTAGTACGCCGGCTTG

### 2.4. DNA Sequencing and Phylogenetic Analyses

PCR-amplified products were sequenced by the Mission Biotech Company (Taipei, Taiwan). The sequences were analyzed in an auto sequencer (Applied Biosystems, Foster City, CA, USA) using a terminator cycler sequencing ready reaction kit (Applied Biosystems, USA). Additional sequences that did not originate from the sequencing of these 12 isolates were downloaded from NCBI. Completed sequences were imported into the program BioEdit sequence alignment editor version 7.0.9.0. [6], and aligned using the CLUSTALW [7] option. Phylogenetic trees were constructed using maximum likelihood analysis in the phylogenetic analysis using parsimony 4.0 program (PAUP 4.0, http://paup.csit.fsu.edu/).

### 2.5. Different Types of nrDNA Copy Number Ratio Analyses

The different types of nrDNA were amplified 10 to 35 cycles, respectively, to estimate copy number ratio. The forward primers GC18NS7 and AT1218NS7 and reverse primers GCITS4, AT12ITS4, and TITS4 (Table 2, Figure 1) pairs were used to amplify ITS1, 5.8S, and ITS2 nrDNA.

## 3. Results

Primer pair NS5/GCITS4 amplified sequences from all six isolates, which yielded products of 1219 bp. Primer pair NS5/AT12ITS4 amplified sequences from isolates Cs7528A-0901 and RS3-0901, yielding products of 1223 bp. NS5/TITS4 was unable to amplify the sequence of any isolate. We replaced this pair with TITS5b/TITS4, and by doing so we were able to successfully amplify ITS region sequences from isolates Korea-0901, W1023-0901, and RS4-2-0901. However, the resulting products were smaller at 477 bp. The GenBank accession numbers of the amplified products are shown in Table 1.

**Figure 2.**
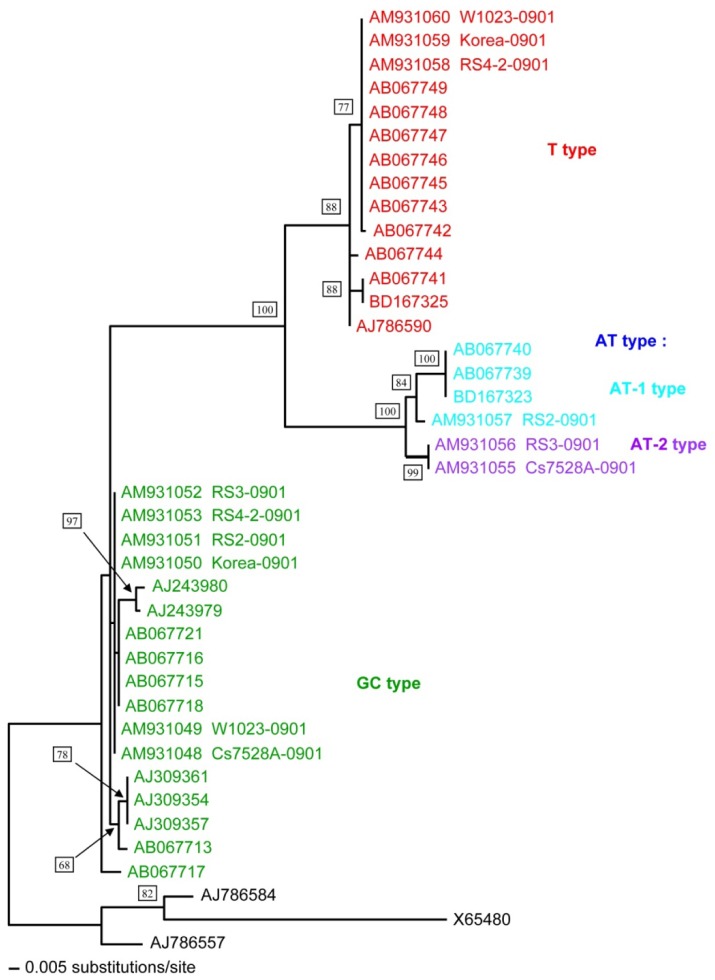
Phylogenetic tree resulting from the Maximum Likelihood Method (ML) of the nrDNA ITS1, 5.8S, ITS2 region sequences of *Ophiocordyceps sinensis* and other related fungi. Bootstrap percentage values of ML are shown at each branch.

Based on the ITS region sequences phylogenetic tree (Figure 2), *O. sinensis* could be divided into three types: GC-type (GC content 64.6%), T-type (GC content 54.4%) and AT-type (GC content 51.4%; this could be further divided into AT-1 and AT-2 types). The sequence identities between each type were between 85.1% and 96.7% (Table 3).

**Figure 3.**
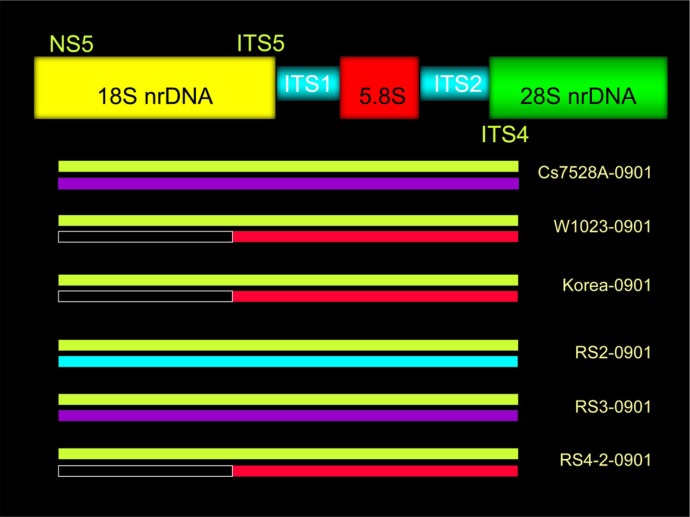
The nrDNA 18S and ITS region types of *Ophiocordyceps sinensis* isolates. The green band indicates the GC-type sequence, the purple band indicates the AT-2 type, and the blue band indicates the AT-1 type. The red band, in which was discovered only the ITS region, indicates the T type.

Crucially, we found that each *O. sinensis* isolate simultaneously had two types of ITS region nrDNA sequences (Table 1, Figure 3): the GC-type sequence and one other sequence type. Analogous results showed that a single *O. sinensis* isolate had two types of 18S nrDNA sequence (partial) simultaneously. Isolate Cs7528A-0901 contained both the GC-type and AT-type sequences, RS2-0901 contained the GC-type and AT-type sequences, and RS3-0901 contained the GC-type and AT-type sequences. The sequence identities between each type were between 84.7% and 98.6% (Table 4).

**Table 3.** Sequence identities between different types of ITS region nrDNA in *Ophiocordyceps sinensis*

nrDNA types	T	AT-1	AT-2	GC
T	1.000	0.898	0.891	0.894
AT-1		1.000	0.967	0.862
AT-2			1.000	0.851
GC				1.000

**Table 4.** Sequence identities between different types of 18S nrDNA (partial) in *Ophiocordyceps sinensis*

nrDNA types	AT-1	AT-2	GC
AT-1	1.000	0.986	0.849
AT-2		1.000	0.847
GC			1.000

The different types of nrDNA were amplified 10 to 35 cycles to estimate copy number ratio. Only primer pairs GC18NS7/GCITS4 (GC-type) and AT1218NS7/AT12ITS4 (AT-type) could successfully amplify ITS1, 5.8S, and ITS2 nrDNA. Other primer pairs, e.g., GC18NS7/AT12ITS4, GC18NS7/TITS4, AT1218NS7/GCITS4, and AT1218NS7/TITS4 failed. For example, in strain RS2-0901, the AT-type first appeared after the 20th cycle and the GC-type first appeared after the 16th cycle, indicating that the GC-type was 16-fold greater than the AT-type.

## 4. Discussion

nrDNA sequences are widely used in the molecular classification of fungi. The primers used in such analyses are mainly designed based on the nrDNA sequences of *Saccharomyces cerevisiae* [5]. By virtue of primer specificity, usually unique-type sequences are amplified and used for phylogenetic analysis. Otherwise, the absence of reference sequences restricts the design of primers, and other types of sequences are difficult to amplify. Therefore, a single species has traditionally been considered to have one nrDNA sequence. According to this concept, most earlier molecular researchers amplified only the GC-type nrDNA sequences and consequently inferred a low sequence divergence within *O. sinensis* [8]–[15]. When different types of nrDNA were amplified in earlier molecular studies, they were interpreted as being subgroups of a “species complex” [2] or as “cryptic species” [3].

In the present study, the *O. sinensis* isolates were indistinguishable from each other on the basis of morphological features. However, each had the constituent GC-type nrDNA as well as other types of nrDNA. This result demonstrated that classification of *O. sinensis* (and other fungal species) based on nrDNA sequences should ideally take into consideration the composition of nrDNA types.

An outstanding question, however, is why isolates Korea-0901, W1023-0901, and RS4-2-0901, which have the T-type ITS region nrDNA sequence, did not have corresponding T-type 18S nrDNA sequences. There are two possible explanations for this disparity. The T-type ITS region nrDNA sequence may correspond to the GC-type 18S nrDNA sequence. Alternatively, the T-type ITS region nrDNA sequence may correspond to the T-type 18S nrDNA sequence, which we have been unable to amplify. Since no amplification products were obtained using the primer pair NS5 (GC-type 18S nrDNA forward primer)/TITS4 (T-type ITS region nrDNA reverse primer), the second explanation may be more plausible.

There are also literary records of single species having more than one nrDNA sequences, including the Ascomycetes *Fusarium graminearum*, *Gibberella fujikuroi* [16],[17], and *Neurospora* [18], the Basidiomycetes *Trichaptum abietinum* [19], various plant species [20], the tiger beetle *Cicindela dorsalis* [21], and the nematode species *Meloidogyne hapla* and *M. chitwoodi* [22]. Notably, the variation of nrDNA in the listed citations only occurs in locations that do not affect mature rRNA function. For example, the two types of nrDNA in *F. graminearum* and *G. fujikuroi* [16],[17] occur mainly in the ITS2 region, and in *T. abietinum* [19], tri-type nrDNA occurred mainly in the ITS1 region, after the formation of pre-rRNA would have been removed. Thus, rRNA and ribosome composition are not affected.

In the present study, we found not only the variation of nrDNA in ITS1 and ITS2, but also that there were multiple types of rRNA and ribosomes in single *O. sinensis* in the functional 18S and 5.8S nrDNA regions. The differences in ribosomes may lead to differences in their synthetic proteins, and may also lead to regionally-sourced *O. sinensis* having different medicinal effects. In addition, intra-individual variation in nrDNA is common, and different copies with different GC-content are frequent. Such AT-rich copies are usually considered non-functional pseudogenes [23]. The low GC content characteristic of many non-recombining genomes may be the result of three processes: (1) a prevailing AT mutational bias; (2) random fixation of the most common types of mutation; and (3) the absence of the GC-biased gene conversion, which, in recombining organisms, permits the reversal of the most common types of mutation [24]. According to this theory, the AT-type and T-type of *O. sinensis* may have experienced a shift in life history towards a prevailingly non-recombining clonal life style, such as the anamorph stage of life cycle [3].

Another hypothesis raised to account for the AT bias observed in obligate biotrophs is that competition for metabolic resources may exist between host and parasite [25]. In *O. sinensis*, this could be due to situations such as a shift towards an even more obligate biotrophic life style in AT-type and T-type, which is the anamorph stage [3]. Further research, including a larger pool of molecular and ecological data on *O. sinensis* isolates in nature and from culture, will be required to resolve the above hypotheses.
